# Wireless wearables for postoperative surveillance on surgical wards: a survey of 1158 anaesthesiologists in Western Europe and the USA

**DOI:** 10.1016/j.bjao.2022.100002

**Published:** 2022-02-23

**Authors:** Frederic Michard, Robert H. Thiele, Bernd Saugel, Alexandre Joosten, Moritz Flick, Ashish K. Khanna, Collaborators, Collaborators in Europe, Matthieu Biais, Vincent Bonhomme, Wolfgang Buhre, Bernard Cholley, Jean-Michel Constantin, Emmanuel Futier, Samir Jaber, Marc Leone, Benedikt Preckel, Daniel Reuter, Patrick Schoettker, Thomas Scheeren, Michael Sander, Luzius A. Steiner, Sascha Treskatsch, Kai Zacharowski, Collaborators in the USA, Anoushka Afonso, Lovkesh Arora, Michael L. Ault, Karsten Bartels, Charles Brown, Daniel Brown, Douglas Colquhoun, Ryan Fink, Tong J. Gan, Neil Hanson, Omar Hyder, Timothy Miller, Matt McEvoy, Ronald Pearl, Romain Pirracchio, Marc Popovich, Sree Satyapriya, B. Scott Segal, George Williams

**Affiliations:** 7 Université de Bordeaux, Hôpital Tripode, Bordeaux, France; 8 Centre Hospitalier Universitaire, Liège, Belgium; 9 University Medical Center, Maastricht, the Netherlands; 10 Université de Paris, INSERM, IthEM, Hopital Européen Georges Pompidou, AP-HP, Paris, France; 11 University Paris-Sorbonne, Hopital Pitié-Salpétrière, Paris, France; 12 Université Clermont Auvergne, Hopital d’Estaing, Clermont-Ferrand, France; 13 Université de Montpellier, Saint Eloi Hospital, Montpellier, France; 14 Université Aix-Marseille, Hopital Nord, Marseille, France; 15 Amsterdam University Medical Centers Location AMC, the Netherlands; 16 Rostock University Medical Center, Rostock, Germany; 17 Université de Lausanne, CHUV, Lausanne, Switzerland; 18 University Medical Center, Groningen, the Netherlands; 19 University Medical Center, Giessen, Germany; 20 Anesthesiology, University Hospital Basel and Department of Clinical Research, University of Basel, Switzerland; 21 Charité University Medical Center, Berlin, Germany; 22 University Medical Center, Frankfurt, Germany; 23 Memorial Sloan Kettering Cancer Center, New York City, NY, USA; 24 University of Iowa Health Care, Iowa City, IA, USA; 25 Northwestern Memorial Hospital, Chicago, IL, USA; 26 University of Nebraska Medical Center, Omaha, NE, USA; 27 Johns Hopkins University Hospital, Baltimore, MD, USA; 28 Mayo Clinic, Rochester, MN, USA; 29 University of Michigan Health, Ann Arbor, MI, USA; 30 Oregon Health Services University, Portland, OR, USA; 31 StonyBrook University Medical Center, StonyBrook, NY, USA; 32 University of Minnesota Medical Center, Minneapolis, MN, USA; 33 Massachussets General Hospital, Harvard Medical School, Boston, MA, USA; 34 Duke University Hospital, Durham, NC, USA; 35 Vanderbilt University Medical Center, Nashville, TN, USA; 36 Stanford University School of Medicine, Stanford, CA, USA; 37 University of California San Francisco, CA, USA; 38 University Hospitals Cleveland Medical Center, Cleveland, OH, USA; 39 Ohio State University, Columbus, OH, USA; 40 Wake Forest University Medical Center, Winston-Salem, NC, USA; 41 University of Texas Medical Center, Houston, TX, USA; 1 MiCo, Denens, Switzerland; 2 Department of Anesthesiology, University of Virginia, Charlottesville, VA, USA; 3 Department of Anesthesiology, Center of Anesthesiology and Intensive Care Medicine, University Medical Center Hamburg–Eppendorf, Hamburg, Germany; 4 Outcomes Research Consortium, Cleveland, OH, USA; 5 Department of Anesthesiology, University Paris Saclay, Paul Brousse Hospital, Villejuif, France; 6 Department of Anesthesiology, Wake Forest School of Medicine, Winston-Salem, NC, USA

**Keywords:** anaesthesiology, failure to rescue, monitoring, patient safety, postoperative complications, surgery, wearables

## Abstract

**Background:**

Several continuous monitoring solutions, including wireless wearable sensors, are available or being developed to improve patient surveillance on surgical wards. We designed a survey to understand the current perception and expectations of anaesthesiologists who, as perioperative physicians, are increasingly involved in postoperative care.

**Methods:**

The survey was shared in 40 university hospitals from Western Europe and the USA.

**Results:**

From 5744 anaesthesiologists who received the survey link, there were 1158 valid questionnaires available for analysis. Current postoperative surveillance was mainly based on intermittent spot-checks of vital signs every 4–6 h in the USA (72%) and every 8–12 h in Europe (53%). A majority of respondents (91%) considered that continuous monitoring of vital signs should be available on surgical wards and that wireless sensors are preferable to tethered systems (86%). Most respondents indicated that oxygen saturation (93%), heart rate (80%), and blood pressure (71%) should be continuously monitored with wrist devices (71%) or skin adhesive patches (54%). They believed it may help detect clinical deterioration earlier (90%), decrease rescue interventions (59%), and decrease hospital mortality (54%). Opinions diverged regarding the impact on nurse workload (increase 46%, decrease 39%), and most respondents considered that the biggest implementation challenges are economic (79%) and connectivity issues (64%).

**Conclusion:**

Continuous monitoring of vital signs with wireless sensors is wanted by most anaesthesiologists from university hospitals in Western Europe and in the USA. They believe it may improve patient safety and outcome, but may also be challenging to implement because of cost and connectivity issues.

Postoperative mortality is a common cause of death the world over,^1^ and “failure to rescue” is considered a major surgical quality indicator.^2^^,^^3^ When patients are in the operating theatre and the PACU they are monitored closely, if not continuously. However, as soon as they are discharged to a post-surgical ward, they move to a place with a much lower nurse/patient ratio and where vital signs are usually checked intermittently. To minimise the number of severe adverse events after surgery and tackle failure to rescue, several publications have recently suggested the need to improve patient surveillance strategies on surgical wards.^4^, ^5^, ^6^, ^7^, ^8^, ^9^, ^10^

Complications occur after almost one-third of major surgical procedures, in patients with significant co-morbidities, or both.^11^ The most common complications are infectious, cardio-vascular, and haemorrhagic.^11^ Patients receiving opioids for postoperative pain management are also exposed to the risk of respiratory depression.^12^ Intermittent measurement of vital signs may lead to the delayed detection of clinical deterioration.^13^, ^14^, ^15^ By enabling the early detection of clinical trajectories,^16^ continuous monitoring of vital signs may help to decrease severe adverse events requiring rescue interventions, ICU admissions, and deaths.^17^, ^18^, ^19^, ^20^, ^21^, ^22^, ^23^, ^24^

Several surveillance systems have recently been approved by regulatory bodies in Europe, the USA, or both, for ward-based continuous monitoring of one or more vital signs. They include ‘stay-in-bed’ solutions,^9^ such as bed sensors and video-cameras, and wireless wearable solutions, such as finger sensors, chest or abdominal adhesive patches, and bioimpedance necklaces.^25^, ^26^, ^27^ Little is known regarding the clinical and economic impact of these new monitoring solutions,^28^ or the challenges to hospital implementation.^29^^,^^30^ Therefore, we designed an international survey to understand the current perception and expectations of anaesthesiologists who, as perioperative physicians, are increasingly involved in postoperative care.^31^, ^32^, ^33^, ^34^

## Methods

The survey was approved by the Ethics Committee for Research in Anaesthesia and Critical Care (CERAR) of the French Society of Anaesthesia and Critical Care (SFAR) on October 17, 2021 (IRB #0010254-2021-195), and the permission to use responses for analysis and publication was asked in the questionnaire. Anonymous English and French electronic questionnaires containing 15 short questions were developed by the authors using Google Forms (see Questionnaire in the Supplementary material). Both questionnaires were tested by the authors and several colleagues before fielding.

The survey link was shared by email with anaesthesiologists from 40 university hospitals (convenience sample for an expected number of respondents >1000) in Western Europe and in the USA by one leadership member of each site (please see list of collaborators in Appendix A). No incentive was offered for participation. A reminder email was sent after 1 or 2 weeks, and the database was locked after 4 weeks. A denominator (number of anaesthesiologists who received the survey link) was provided by each site to enable the calculation of a global response rate.

Questionnaires not completed by an anaesthesiologist (board-certified or resident), that did not contain country information, or with more than three unanswered questions were considered invalid. When respondents did not give us the permission to use their responses for publication (last question of the survey), questionnaires were also excluded from analysis. Data are presented as numbers and percentages. Multiple answers were allowed for several questions (see Questionnaire in the Supplementary material) so that combined percentages presented in the text or the figures may exceed 100%. Because respondents could leave individual questions unanswered, all reported proportions are relative to the number of responses to each question. Comparisons between Western Europe and the USA were made with a χ^2^ test. To reduce the chance of false-positive findings, we set the statistical significance level at *P*<0.01. Results are reported according to published recommendations to improve the quality of electronic surveys.^35^

## Results

**Fig 1:**
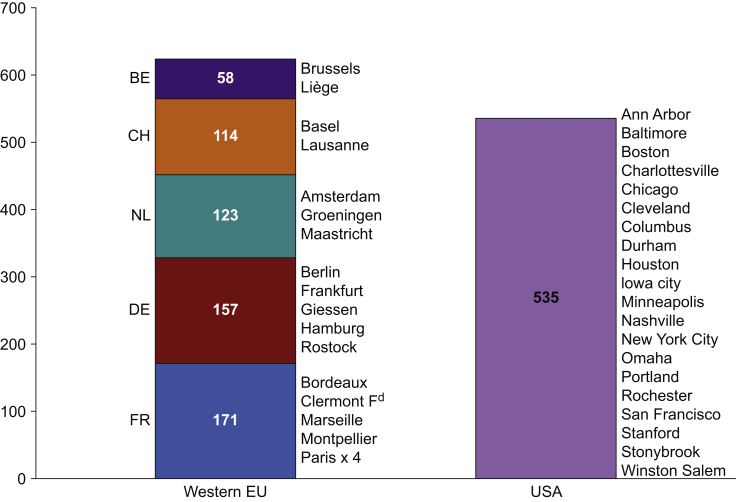
Number and origin of valid questionnaires. EU, Europe; BE, Belgium; CH, Switzerland; NL, Netherlands; DE, Germany; FR, France.

The survey link was shared with 5744 clinicians from 20 anaesthesia departments in Western Europe (eight in France, five in Germany, three in the Netherlands, two in Belgium, and two in Switzerland) and 20 in the USA (Fig. 1). The survey link was shared from week 42 and the survey database was locked for analysis at the end of week 46 after receiving 1196 responses (response rate 21%). Twenty questionnaires were filled by non-anaesthesiologists, one did not contain the country information, and another contained more than three unanswered questions. In addition, 16 respondents did not give us the permission to use their answers for analysis and publication. Therefore, 1158 questionnaires were finally available for analysis, 623 (54%) from Western Europe and 535 (46%) from the USA (Fig. 1).

Most respondents (57%) were board-certified anaesthesiologists working mainly in the operating theatre and 30% were residents with at least 1 yr experience. Current postoperative surveillance was mainly based on intermittent spot-checks of vital signs, every 4–6 h (two times per nurse shift) in the USA (72%), and every 8–12 h (one time per nurse shift) in Europe (53%). Continuous monitoring with tethered systems may be used occasionally according to 40% of respondents, and more frequently in the USA than in Europe (53% *vs* 29%, *P*<0.01). Continuous monitoring with wireless systems may be used occasionally according to a small proportion of respondents (14%), more frequently in the USA than in Europe (23% *vs* 6%, *P*<0.01).

A majority of respondents considered that automatic and continuous monitoring of vital signs should be available on surgical wards (91%), that mobile solutions with wireless sensors were preferable to tethered solutions (86%) and that only patients at high risk of clinical deterioration should be monitored continuously (73%). Most respondents believed that automatic and continuous monitoring of vital signs may help to detect clinical deterioration earlier (90%), to decrease Rapid Response Team (RRT) interventions on the wards (59%) and hospital mortality (54%). They thought that patients who may benefit the most are those recovering from cardiac surgery (80%), thoracic surgery (75%), general or abdominal surgery (70%), and neurosurgery (51%).

**Fig 2:**
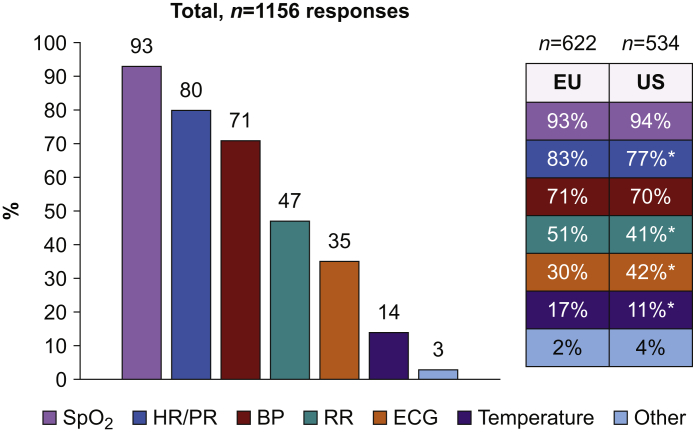
Which variables would you monitor continuously? ∗*P*<0.01 for comparison between Western Europe (EU) and the USA (US). SpO_2_, oxygen saturation; HR/PR, heart rate/pulse rate; RR, respiratory rate.

**Fig 3:**
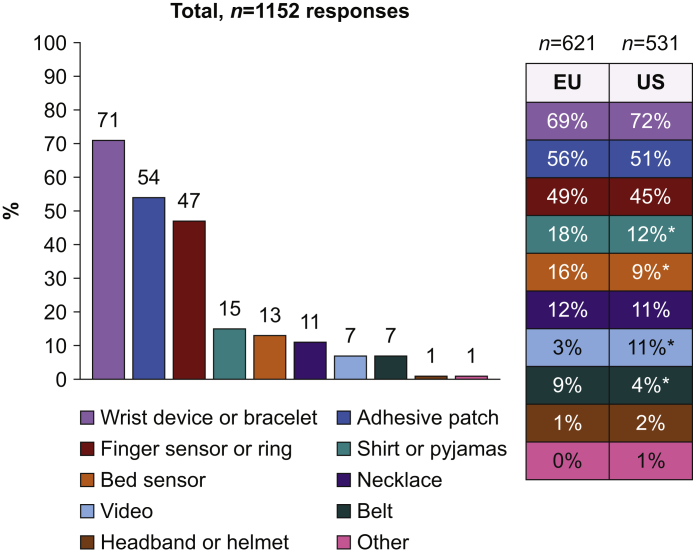
Which sensor would be ideal for continuous monitoring of vital signs? ∗*P*<0.01 for comparison between Western Europe (EU) and the USA (US).

Of vital signs that should be continuously monitored, oxygen saturation was supported by 93% of respondents, heart rate by 80%, and blood pressure 71% (Fig. 2). Almost half of respondents (47%) recommended continuous respiratory rate monitoring, 35% recommended ECG monitoring, and only 14% temperature monitoring (Fig. 2). Wrist devices or bracelets (71% of respondents) and skin adhesive patches (54% of respondents) were the preferred patient interface (Fig. 3). Finger sensors or rings were considered as potentially ideal by 47% of respondents, whereas sensors in textile (pyjamas), bed sensors, and necklaces were selected by 15%, 13%, and 11% of respondents, respectively (Fig. 3).

**Fig 4:**
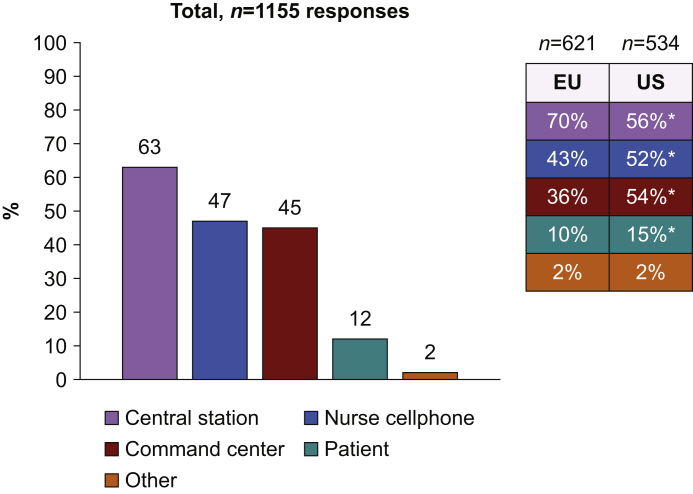
Where alarms should be heard and/or seen? ∗*P*<0.01 for comparison between Western Europe (EU) and the USA (US).

Regarding alarms, 63% of respondents preferred that alarms should be heard or seen at a central station on the wards. Most respondents from the USA considered that alarms could also be sent directly to the nurse or to a command centre with dedicated staff. Overall, only 12% of respondents thought that alarms should be heard or seen by patients (Fig. 4).

Opinions diverged regarding the impact of automatic and continuous monitoring on nurse workload (increase 46%, decrease 39%) and hospital costs (decrease 39%, increase 34%). Most respondents considered that the biggest implementation challenges are the economic aspect (79%), connectivity issues (64%), and nurse pushback (58%).

## Discussion

According to most respondents, postoperative surveillance is currently based on intermittent spot-checks of vital signs, every 4–6 h in the USA and every 8–12 h in Europe. Interestingly, 91% of anaesthesiologists working in university hospitals from Western Europe and the USA considered that automatic and continuous monitoring of vital signs should be available on surgical wards. In patients with significant co-morbidities or those who underwent a major procedure, close surveillance may be prolonged on occasion beyond the PACU with tethered pulse oximeters, capnography, ECG telemetry, or a combination of these. These monitoring systems are somewhat obtrusive for patients and do not facilitate early mobilisation, which is a key element of enhanced recovery programmes. This may explain why 86% of respondents preferred mobile wireless monitoring solutions.

Most respondents considered that continuous monitoring enables the earlier detection of clinical deterioration, a decrease in RRT interventions, and in-hospital mortality. These findings are consistent with the results of at least seven large (>1000 patients) studies reporting significant postoperative clinical outcome benefits after the implementation of continuous monitoring on hospital wards.^17^, ^18^, ^19^, ^20^, ^21^, ^22^, ^23^ Five of these studies^17^^,^^19^^,^^21^, ^22^, ^23^ reported a significant decrease in the number of RRT interventions after the implementation of continuous monitoring of heart rate, respiratory rate, blood pressure, and/or oxygenation saturation on regular wards. Two studies^17^^,^^23^ reported a significant decrease in the number of ICU transfers. In one study,^20^ the continuous monitoring of heart rate, oxygen saturation, and respiratory rate in 2263 ward patients was associated with a significant decrease in the number of cardiac arrests and in hospital mortality.

Almost three-quarters of respondents considered that the use of continuous monitoring systems should be limited to surgical patients at high risk of postoperative clinical deterioration. This finding highlights the need for risk stratification tools. Several solutions have been proposed to predict clinical deterioration on regular wards, from simple early warning scores (e.g. National Early Warning Score [NEWS]) to logistic regression-derived scores^36^^,^^37^ (e.g. Surgical Outcome Risk Tool [SORT]) and scores obtained from machine learning algorithms^38^^,^^39^ (e.g. electronic Cardiac Arrest Risk Triage [eCART]). The abovementioned scores have different advantages and limitations, but they are all prone to help select patients who may benefit the most from continuous monitoring and hence to rationalise the implementation of new monitoring solutions on hospital wards.

Among vital signs to be monitored continuously, most respondents prioritised oxygen saturation and heart rate. These variables are easy to monitor with skin surface electrodes and/or pulse oximetry, and a large study (402 023 RRT activations) including 360 US hospitals showed that abnormal oxygen saturation, heart rate, or both are the most common RRT activation triggers.^40^ Continuous monitoring of blood pressure was also selected by more than two-thirds of respondents. Recent studies^14^^,^^15^ have demonstrated that postoperative hypotension is common and may go undetected for hours. The association between hypotension after surgery and postoperative morbidity and mortality has been shown to be much stronger than hypotension during surgery.^41^^,^^42^ Therefore, several solutions have emerged for the continuous monitoring of blood pressure on hospital wards. They are based on pulse wave transit time (or pulse arrival time) or on pulse decomposition methods.^43^ In the future, machine learning algorithms may enable the computation of blood pressure from pulse oximetry waveforms.^43^ Continuous respiratory rate monitoring was recommended by less than half of respondents. This is surprising because respiratory rate has been shown to be a very sensitive marker of clinical deterioration,^38^^,^^44^ and multiple new methods have been proposed for the automatic and continuous monitoring of respiratory rate,^9^ from wireless impedance pneumography^45^ to video monitoring^46^ and adhesive patches containing acoustic or piezo-electric sensors and/or accelerometers.^27^

For wireless sensors, most respondents preferred wrist devices or bracelets and adhesive patches. Although we clearly mentioned in the relevant question that sensor accuracy had to be assumed, disruptive solutions such as sensors in textiles (pyjamas) or video-monitoring were selected by a small proportion of respondents (Fig. 3). Bed sensors that are able to monitor heart rate and respiratory rate were approved for medical use several years ago, and a large outcome study reported a significant reduction in the number of calls for cardiac arrest after ward implementation.^18^ However, bed sensors were recommended by only 13% of respondents, possibly because they do not function during mobilisation.

Whatever the monitoring system, according to most respondents, alarms should be heard or seen at a central station on surgical wards. In the USA, most respondents also considered alarms could be sent directly to the nurse or to a command centre with dedicated staff. Command centres have emerged in a few US hospitals to optimise care efficiency and patient flow. They may also be used to centralise alarms from hospital wards and trigger the appropriate response.^47^ Interestingly, only 12% of respondents thought that alarms should be heard by patients. Alarms have been designed to alert clinicians to patient deterioration. Ideally, they should not disturb patients and increase their level of stress. Sleep disorders and noises are a common complaint among hospitalised patients.^48^ This important feedback highlights the fact that modern monitoring systems should be able to alert caregivers exclusively, allowing patients to recover in a quiet and peaceful environment.

There was clearly no consensus regarding the impact of continuous monitoring on hospital costs and nurse workload, but both the economic impact and nurse pushback were considered major challenges to clinical implementation. Costs related to monitoring equipment may be balanced by savings related to a decrease in severe adverse events responsible for a prolonged hospital stay or ICU admission. Modelling^49^ and local evaluations^50^ have suggested economic benefits, but large prospective studies are warranted to clarify the cost-effectiveness of continuous ward monitoring. Similarly, the impact on nurse workload may be twofold. On one hand, the automatic measurement of vital signs has potential to relieve nurses from time consuming and repetitive tasks. On the other hand, additional alarms – and in particular false alarms – may increase nurse workload. This is possibly the reason why most respondents mentioned nurse pushback as one of the main challenges to clinical implementation. Studies are needed to investigate whether the mere increase in annunciation delays (the time interval between the detection of a vital sign abnormality and the alarm), alarm personalisation, and artifact filtering with smart algorithms may help to minimise alarm burden.^51^^,^^52^ It would also be interesting to gather direct feedback from nurses working on surgical wards before speculating on their opinion and potential pushback.

For about two-thirds of respondents, connectivity issues were seen as a significant obstacle to hospital implementation. Bluetooth is frequently used for home monitoring to connect wearable sensors to patients' smartphones. However, disruptions have been shown to be very common and do not allow truly continuous monitoring.^53^ Bluetooth connectivity may not be robust enough to secure continuous monitoring and safety of inpatients.^54^ Therefore, medical grade connectivity protocols may be needed for the successful implementation of mobile monitoring on the wards.

Our study has several limitations. We have been able to collect feedback from >1000 anaesthesiologists working in leading institutions. However, the medical centres we surveyed were all academic institutions and were not randomly selected. They belonged to the authors' network so that our findings may not be representative of the current perception and expectations of all anaesthesiologists working in Western Europe and the USA. Although we used a top–down approach (the survey link was sent by a leadership team member from each site), the response rate was 21%. This may reflect, at least in part, the fact that many anaesthesiologists are not involved or may not be interested in postoperative care, a key component of perioperative medicine.^31^, ^32^, ^33^, ^34^ In addition to the monitoring of vital signs, clinical examination, urine output, and biological data (e.g. troponin, creatinine, haemoglobin, and lactate concentrations) may be useful to detect postoperative complications. Machine learning algorithms fed by clinical and biological data have been shown to be useful to predict clinical deterioration at an early stage.^38^^,^^39^ They were not mentioned in the survey that was intentionally kept short with a focus on vital signs monitoring. Finally, it is important to emphasise that our study remains a survey of anaesthesiologists' opinion. In this respect, clinical studies are necessary to know whether the real-world implementation of continuous and mobile monitoring systems matches the current perception and expectations of practising anaesthesiologists.

## Conclusion

Automatic and continuous monitoring of vital signs with wireless sensors is perceived to be useful by a large majority of anaesthesiologists from university hospitals in Western Europe and in the USA. Most believe it may improve patient safety and outcome. Opinions diverge regarding the impact on nurse workload. Both hospital costs and connectivity issues are considered the biggest challenges to hospital implementation.

## Author's contributions

Study conception, data analysis and manuscript draft: FM.

Questionnaire design: FM, BS, MF.

Site selection and contact: FM, RHT, BS, AJ, AKK.

Ethical committee submission: FM, AJ.

Revising paper: all authors.

All authors approved the final version of the paper.

## Funding

The authors did not receive any funding for the present study.

## Appendix A List of collaborators

### Collaborators in Europe

Matthieu Biais, Université de Bordeaux, Hôpital Tripode, Bordeaux, France.

Vincent Bonhomme, Centre hospitalier universitaire, Liège, Belgium.

Wolfgang Buhre, University Medical Center, Maastricht, the Netherlands.

Bernard Cholley, Université de Paris, INSERM, ithEM, Hopital Européen Georges Pompidou, AP-HP, Paris, France.

Jean-Michel Constantin, University Paris–Sorbonne, Hopital Pitié-Salpétrière, Paris, France.

Emmanuel Futier, Université Clermont Auvergne, Hopital d’Estaing, Clermont-Ferrand, France.

Samir Jaber, Université de Montpellier, Saint Eloi Hospital, Montpellier, France.

Marc Leone, Université Aix-Marseille, Hopital Nord, Marseille, France.

Benedikt Preckel, Amsterdam University Medical Centers Location AMC, The Netherlands.

Daniel Reuter, Rostock University Medical Center, Rostock, Germany.

Patrick Schoettker, Université de Lausanne, CHUV, Lausanne, Switzerland.

Thomas WL Scheeren, University Medical Center Groningen, Groningen, the Netherlands.

Michael Sander, University Medical Center, Giessen, Germany.

Luzius A Steiner, Anesthesiology, University Hospital Basel and Department of Clinical Research, University of Basel, Switzerland.

Sascha Treskatsch, Charité University Medical Center, Berlin, Germany.

Kai Zacharowski, University Medical Center, Frankfurt, Germany.

### Collaborators in the USA

Anoushka Afonso, Memorial Sloan Kettering Cancer Center, New York City, NY.

Lovkesh Arora, University of Iowa Health Care, Iowa city, IA.

Michael L Ault, Northwestern Memorial Hospital, Chicago, IL.

Karsten Bartels, University of Nebraska Medical Center, Omaha, NE.

Charles Brown, Johns Hopkins University Hospital, Baltimore, MD.

Daniel Brown, Mayo Clinic, Rochester, MN.

Douglas Colquhoun, University of Michigan Health, Ann Arbor, MI.

Ryan Fink, Oregon Health Services University, Portland, OR.

Tong J Gan, StonyBrook University Medical Center, StonyBrook, NY.

Neil Hanson, University of Minnesota Medical Center, Minneapolis, MN.

Omar Hyder, Massachussets General Hospital, Harvard Medical School, Boston, MA.

Timothy Miller, Duke University Hospital, Durham, NC.

Matthew D McEvoy, Vanderbilt University Medical Center, Nashville, TN.

Ronald G Pearl, Stanford University School of Medicine, Stanford, CA.

Romain Pirracchio, University of California San Francisco, CA.

Marc Popovich, University Hospitals Cleveland Medical Center, Cleveland, OH.

S Veena Satyapriya, Ohio State University, Columbus, OH.

B. Scott Segal, Wake Forest School of Medicine, Winston–Salem, NC.

George Williams, UTHealth McGovern Medical School, Houston, TX.
