# Supplementary material for: Wireless wearables for postoperative surveillance on surgical wards: a survey of 1158 anaesthesiologists in Western Europe and the USA
Source: BJA Open. 2022 Feb 23;1:100002. doi: 10.1016/j.bjao.2022.100002 (PMC10430871; doi:10.1016/j.bjao.2022.100002)
Supplement: Multimedia component 1 [file mmc1.docx]

**Supplementary data: Questionnaire (English version)**

THANKS for taking 3 mins of your time to answer the following questions.

Please understand that when we mention “SURGICAL WARDS” we refer to the surgical wards of YOUR hospital and EXCLUDE intensive care units (ICUs) and post-anesthesia care units (PACUs)

**Questions:**

1. **Who are you?**
   1. Certified anesthesiologist working mainly in the OR
   2. Certified anesthesiologist working mainly in the ICU
   3. Resident in anesthesiology with at least 1 yr experience
   4. Other
2. **Where are you based?**
   1. Belgium
   2. France
   3. Germany
   4. Switzerland
   5. The Netherlands
   6. USA
3. **MULTIPLE CHOICE QUESTION: On surgical wards (ICU and PACU excluded), vital signs (heart rate, blood pressure, temperature, SpO_2_, and/or respiratory rate)**
   1. Are usually measured every 4-6h (2 times per nurse shift)
   2. Are usually measured every 8-12h (1 time per nurse shift)
   3. May be continuously monitored with one or more tethered (wired) monitoring systems (for example a pulse oximeter)
   4. May be continuously monitored with one or more wireless sensors
   5. Other
4. **MULTIPLE CHOICE QUESTION: Assuming you had the opportunity to monitor vital signs automatically and continuously on surgical wards, do you think it may:**
   1. Detect clinical deterioration earlier compared to intermittent spot-checks
   2. Decrease the number of Rapid Response Team/Medical Emergency Team interventions (including CPR for cardiac arrest)
   3. Decrease the number of ICU admissions
   4. Decrease hospital mortality
   5. None of the above
5. **MULTIPLE CHOICE QUESTION: Assuming you had the opportunity to monitor vital signs automatically and continuously on surgical wards, do you think it may:**
   1. Decrease nurse workload (for example by decreasing the time spent to measure vital signs manually)
   2. Increase nurse workload (for example because of alarms)
   3. I do not know
6. **MULTIPLE CHOICE QUESTION: Assuming you had the opportunity to monitor vital signs automatically and continuously on surgical wards, do you think it may:**
   1. Decrease hospital costs (for example by decreasing ICU transfers)
   2. Increase hospital costs (for example because of the equipment and training required)
   3. I do not know
7. **MULTIPLE CHOICE QUESTION: Assuming you had the opportunity to monitor vital signs automatically and continuously on surgical wards, where do you think it would help the most:**
   1. General or abdominal surgical ward
   2. Orthopedic surgical ward
   3. Thoracic surgical ward
   4. Cardiac surgical wards
   5. Neurosurgical wards
   6. Nowhere
   7. Other
8. **MULTIPLE CHOICE QUESTION: Assuming you had the opportunity to monitor vital signs automatically and continuously on surgical wards, which vital signs would you monitor?**
   1. Heart rate or pulse rate
   2. ECG (1 lead or more)
   3. Oxygen saturation from pulse oximetry (SpO2)
   4. Respiratory Rate
   5. Blood pressure
   6. Temperature
   7. I would not be interested in monitoring automatically and continuously any vital sign
   8. Other
9. **Assuming you had the opportunity to monitor vital signs automatically and continuously, what do you think the monitoring strategy should be:**
   1. All patients admitted to the surgical ward should be monitored continuously
   2. Only patients at high-risk of clinical deterioration should be monitored continuously
   3. I would not be interested in monitoring automatically and continuously any vital sign
10. **MULTIPLE CHOICE QUESTION: Assuming accuracy has been established, what kind of sensor would be ideal for the automatic and continuous monitoring of vital signs**
    1. Video monitoring (video camera watching the patient)
    2. Bed sensor (under the mattress or part of the mattress)
    3. Skin adhesive patch (on the thorax, the abdomen or elsewhere)
    4. Wrist device or bracelet
    5. Finger sensor or ring
    6. Necklace
    7. Headband or helmet
    8. Belt
    9. Shirt or pajama (sensor in textile)
    10. other
11. **For the automatic and continuous monitoring of vital signs, do you think**
    1. Mobile solutions with wireless sensors are preferable so that patients remain monitored when they leave their bed (for example to go to the bathroom) or their room (for example for physiotherapy in the corridor)
    2. Mobile solutions are not indispensable since most patients who require continuous monitoring will stay in bed anyway
    3. I do not know
12. **MULTIPLE CHOICE QUESTION: Regarding alarms, do you think**
    1. alarms should be seen or heard by the patient
    2. alarms should be received on the pager or cellphone of the nurse so that she/he is immediately informed when a patient is deteriorating
    3. alarms should be seen or heard at a central station on the wards
    4. alarms should be centralized in a dedicated command center with dedicated staff who would inform the responsible nurse or doctor or the RRT/MET in case of deterioration
    5. Other
13. **MULTIPLE CHOICE QUESTION: What could be the biggest challenge(s) for the implementation of automatic and continuous monitoring systems on surgical wards**
    1. Patient pushback
    2. Nurse pushback
    3. Ward physician pushback
    4. The economic aspect (administration pushback)
    5. Connectivity/IT issues
    6. Other
14. **Do you think the automatic and continuous monitoring of vital signs should become available on surgical wards**
    1. Yes
    2. No
15. **FINAL QUESTION: Do you give us the permission to use your answers/anonymous data for analysis and publication**
    1. Yes
    2. No
